# Novo Efeito Cardioprotetor da L-Carnitina em Camundongos Obesos Diabéticos: Regulação da Expressão de Quemerina e CMKLRI no Coração e Tecidos Adiposos

**DOI:** 10.36660/abc.20200044

**Published:** 2021-10-06

**Authors:** Rezvan Amiri, Mohammad Reza Tabandeh, Seyed Ahmad Hosseini

**Affiliations:** 1 Department of Basic Sciences Faculty of Veterinary Medicine Shahid Chamran University of Ahvaz Ahvaz Irã Department of Basic Sciences, Division of Biochemistry and Molecular Biology, Faculty of Veterinary Medicine, Shahid Chamran University of Ahvaz, Ahvaz – Irã; 2 Department of Nutrition Science Ahvaz Jundishapur University of Medical Sciences Ahvaz Irã Department of Nutrition Science, Ahvaz Jundishapur University of Medical Sciences, Ahvaz – Irã

**Keywords:** Diabetes Mellitus, Carnitina, Quimiocinas

## Abstract

**Fundamentos:**

A L-carnitina (LC) tem muitos efeitos benéficos em animais diabéticos e humanos, mas seu efeito regulatório sobre a quemerina como uma citocina inflamatória e seu receptor no estado diabético são desconhecidos.

**Objetivos:**

O presente estudo teve como objetivo investigar o efeito regulatório da LC na expressão do receptor semelhante ao de quimiocina 1 e quemerina (CMKLRI) em tecidos adiposo e cardíaco de camundongos diabéticos.

**Métodos:**

Sessenta camundongos NMARI foram divididos em quatro grupos, incluindo controle, diabético, diabético + suplementação com LC e controle + suplementação com LC. O diabetes foi induzido pela alimentação dos animais com dieta hipercalórica por 5 semanas e injeção de estreptozotocina. Os animais foram tratados com 300 mg/kg de LC por 28 dias. Nos dias 7, 14 e 28 após o tratamento, os níveis de mRNA e proteína da quemerina e CMKLRI nos tecidos cardíacos e adiposos de animais foram determinados utilizando análise por qPCR e ELISA. Os índices de resistência à insulina também foram medidos em todos os grupos experimentais. A diferença com p<0,05 foi considerada significativa.

**Resultados:**

A expressão de quemerina e CMKLRI aumentou nos tecidos cardíaco e adiposo de camundongos diabéticos nos dias 14 e 28 após a indução do diabetes, concomitantemente com a incidência de resistência à insulina e níveis aumentados de quemerina circulante (p<0,05). O tratamento com LC causou uma diminuição significativa na expressão de ambos os genes nos tecidos estudados e redução dos sintomas de resistência à insulina e dos níveis séricos de quemerina (p<0,05).

**Conclusão:**

Os resultados sugerem que o tratamento com LC pode diminuir a expressão de quemerina e CKLR1 em tecidos cardíacos e adiposos de animais experimentais obesos e diabéticos.

## Introdução

Um número crescente de evidências tem mostrado a existência de uma relação bioquímica e molecular complexa e multifacetada entre diabetes mellitus (DM), obesidade e doenças cardiovasculares (DCV).^1^ Um possível mecanismo que liga DM e obesidade com DCV subsequentes é a inflamação de baixo grau em tecido adiposo.^2^ O tecido adiposo de animais ou humanos com DM e resistência à insulina secreta uma variedade de citocinas ou adipocinas. A secreção anormal dessas adipocinas contribui para o aumento da inflamação e do acúmulo de lípides e pode levar ao desenvolvimento de disfunção endotelial e miocárdica e cardiomiopatias.^2 , 3^ As alterações de expressão ou secreção de adipocinas pró-inflamatórias e anti-inflamatórias em cardiomiócitos e tecido adiposo têm sido implicadas na patogênese da síndrome metabólica e DCV em animais ou humanos obesos.^2^

A quemerina é uma adipocina recentemente identificada que atua como uma proteína quimioatrativa e medeia seus efeitos através de um receptor acoplado à proteína G, o receptor semelhante ao de quimiocina 1 (CMKLRI, do inglês *chemokine like receptor 1* ), também conhecido como ChemR23.^4 - 7^ Estudos anteriores demonstraram que a quemerina tinha um papel em várias patologias cardiovasculares, incluindo o desenvolvimento de hipertensão, progressão de lesões ateroscleróticas e função cardíaca prejudicada em pacientes com cardiomiopatia dilatada.^8^ A quemerina pode causar inflamação endotelial e induzir apoptose em cardiomiócitos murinos, demonstrando o seu papel potencial nas disfunções dos cardiomiócitos.^9 , 10^

Recentemente, nutrientes que têm como alvo as mitocôndrias, como o ácido lipóico, a L-carnitina (LC), a nicotinamida e a biotina, receberam maior atenção na melhora das complicações cardiovasculares e inflamatórias relacionadas ao diabetes.^11 , 12^ A L-carnitina (LC) é condicionalmente um nutriente essencial que facilita o transporte do ácido graxo para os locais de oxidação na mitocôndria.^13 , 14^

Há evidências crescentes de que a suplementação de LC pode ser benéfica no tratamento da resistência à insulina e distúrbios metabólicos relacionados à obesidade em pacientes diabéticos.^11^ Vários estudos têm demonstrado que a LC tem efeitos benéficos na prevenção e melhora de doenças cardiovasculares, incluindo insuficiência cardíaca crônica, infarto agudo do miocárdio anterior, doença arterial coronariana, remodelação miocárdica e fibrilação atrial.^12 , 14^ Vários mecanismos têm sido relatados sobre o papel protetor da LC nas células cardíacas sob condição hiperglicêmica, incluindo melhora da homeostase da energia cardíaca, atenuação do estresse oxidativo e dano celular hipóxico e redução da apoptose.^15 , 16^ Evidências limitadas também mostraram que a LC pode afetar a concentração sérica de quemerina em crianças obesas.^17^ Com base em nosso conhecimento, poucos estudos focaram no papel da suplementação de LC na regulação da expressão de quemerina, em particular nos tecidos cardíacos e adiposos de animais diabéticos. O objetivo do presente estudo foi investigar os efeitos da suplementação de LC na expressão de quemerina e CMKLRI em tecido adiposo e tecido cardíaco de um modelo experimental de obesidade e diabetes induzidos por dieta hipercalórica em camundongos.

## Materiais e métodos

### Animais

Sessenta camundongos NMARI machos (25 ± 2 g) foram selecionados do centro de animais de laboratório da faculdade de medicina veterinária da Shahid Chamran University de Ahvaz, Irã. Todos os camundongos foram mantidos em condições ambientais controladas, com temperatura média de 23 ± 1°C, ciclo de luz e escuridão de 12:12 horas, com livre acesso a água e dieta especial de camundongos preparada em pellets (Pars, Teerã, Irã). Todos os protocolos experimentais foram aprovados pelo comitê de ética para pesquisa em animais e humanos da Shahid Chamran University de Ahvaz (EE/98.24.3.26209/SCU.AC.IR). O trabalho com os camundongos foi realizado com base nas diretrizes para o cuidado e uso de animais de laboratório (publicação NIH nº 86-23). Eles foram aclimatados por 7 dias antes do início do experimento.

### Desenho experimental

Os animais (n = 60) foram divididos de forma aleatória em quatro grupos igualmente. A randomização foi realizada por meio da alocação de animais do mesmo sexo, idade e peso em diferentes grupos experimentais através de tabelas de números aleatórios. Dois grupos foram alimentados com dieta rica em energia, preparada com adição de 20% de sacarose (p/p) e 10% de banha (p/p) nas dietas por 5 semanas e descritas como tendo alto teor de gordura/hipercalóricas (HF/HC) (n = 30), enquanto os demais continuaram a consumir dietas normais pelo mesmo período, servindo como grupo controle (n = 30). Após 5 semanas de alimentação com dietas HF/HC, os grupos diabéticos foram tratados com uma única injeção intraperitoneal de estreptozotocina (STZ, Sigma, Alemanha) (30 mg/kg de peso corporal) preparada em solução tampão de citrato.^18 , 19^

A glicose foi medida cinco dias após o tratamento com STZ utilizando um glicosímetro portátil (Medisign, China), e a incidência de diabetes foi confirmada se a glicose sérica estivesse acima de 6,5 mmoL/L e o índice HOMA-IR estivesse aumentado em comparação com o grupo de controle.^20^ O dia seguinte após a confirmação do diabetes foi considerado o dia 0 do tratamento com LC. Um grupo diabético foi tratado com LC na dose de 300 mg/kg (n = 15) em água potável concomitante com a dieta HF/HC por 28 dias, enquanto outro grupo diabético (n = 15) (controle diabético) foi alimentado apenas com a dieta HF/HC pelo mesmo período.^18 , 19^ Também foram considerados um grupo controle (n = 15) que recebeu dieta normal e outro grupo controle (n = 15) (controle tratado com LC) que consumiu LC na dose de 300 mg/kg em água potável por 28 dias.

### Amostragem

A eutanásia dos animais foi realizada com uma combinação de cetamina (100 mg/kg) e xilazina (10 mg/kg), respectivamente, nos dias 0, 14 e 28 após o tratamento com LC. Amostras de sangue foram coletadas e os soros separados e armazenados a -20°C para utilização. O coração e o tecido adiposo visceral foram separados e mantidos a –70°C até a sua utilização.

### Medidas de parâmetros bioquímicos

Os níveis séricos de insulina e quemerina foram determinados com os kits ELISA específicos para a espécie (EastBiopharm, China). A glicose sérica foi medida com kits comerciais (PishtazTeb, Irã) de acordo com a recomendação do fabricante. As concentrações séricas de IL1-β e TNF-α foram determinadas utilizando kits ELISA específicos para a espécie, conforme recomendado pelo fabricante (Biovision Inc. EUA)

### Estimativa de HOMA – IR

O índice HOMA-IR foi calculado utilizando a seguinte fórmula:

Índice HOMA = Insulina em jejum (µU/ml) × Glicose em jejum (mmol/L)/22,5. Valores crescentes e decrescentes de HOMA-IR em relação ao controle, animais saudáveis indicam aumento e diminuição na sensibilidade à insulina, respectivamente.^20^

### Isolamento de RNA e síntese de cDNA

O RNA total foi extraído de 100 mg de coração e tecidos adiposos utilizando o reagente de isolamento RNX^®^ de acordo com o procedimento do fabricante (SinaClon, Teerã, Irã). As amostras foram tratadas com a enzima DNase I para evitar contaminação do DNA. A pureza do RNA na razão 260/280 OD e a integridade do RNA foram avaliadas com uma célula de medição de microvolume em Eppendorf µCuvette G1.0 (Eppendorf BioPhotometer D30, Eppendorf, Alemanha). A transcrição reversa foi realizada com o kit de síntese de cDNA YTA (Yekta tajhiz, Irã) e Eppendorf Thermal Cycler (Alemanha) utilizando 1 µg de RNA e hexâmero aleatório, conforme recomendado pelo fabricante.

### PCR quantitativa em tempo real (RT-PCR)

A reação em cadeia da polimerase (PCR, do inglês *Polymerase Chain Reaction* ) em tempo real foi realizada utilizando o sistema de detecção Roche Light-Cycler (Basel, Suíça) pelo qPCR^®^ Green Master Kit para SYBR Green I^®^ (Yektatajhiz, Irã). O nível de expressão relativa dos transcritos de quemerina e CMKLRI foram comparados com os de camundongos GAPDH como gene *housekeeping* . Os conjuntos específicos de primers (Pishgam BioTech, Co, Teerã, Irã) projetados para este estudo foram: quemerina (GenBank:NC-007299): 5’- TCTTCACCTACGACCAGTATCAG -3’ e 5’- ACATTATCTGCATAGACCCCATTG -3’ e *CMKLRI* (GenBank:NM-008153.3): 5’- GTACGACGCTTACAACGACT -3’ e 5’- GCACACCAAGCTGTAGATCA -3’, *GAPDH* (GenBank:NM-001034055): 5’- CTCATCTACCTCTCCATCGTCTG -3’ e 5’- CCTGCTCTTGTCTGCCGGTGCTTG -3’. O protocolo de PCR utilizado consistiu em uma desnaturação de 5 min a 94°C, seguida por 45 ciclos de 94^o^C por 15 s, 60^o^C por 30 s. Duas reações separadas sem cDNA ou com RNA foram realizadas em paralelo como controles. A quantificação relativa foi realizada de acordo com o método comparativo 2^-^ utilizando o software Lightcycler 96^®^. A validação do ensaio para verificar se os primers para quemerina e CMKLRI e GAPDH apresentavam eficiências de amplificação semelhantes foi realizada conforme descrito anteriormente. Todas as análises por qPCR foram realizadas de acordo com a diretriz *The Minimum Information for Publication of Quantitative Real-Time PCR Experiments* (MIQE).^21^

### Determinação da proteína quemerina tecidual

Os tecidos cardíacos e adiposos foram homogeneizados em 500 μL de tampão de lise RIPA (NaCl; 150 mM, SDS 0,1%, Tris; 25 mM, pH 7,4, NaF; 1 mM, fluoreto de fenilmetilsulfonila 1 mM) com um homogeneizador (Heidolph, Schwabach, Alemanha). O homogenato foi centrifugado a 10.000 × RPM por 15 min a 4°C (Centrífuga 5415 R; Eppendorf AG, Hamburgo, Alemanha). O sobrenadante foi recolhido e armazenado a -70°C para análise subsequente. A concentração de proteína do sobrenadante foi estimada com o método de Bradford. A concentração de quemerina foi determinada utilizando os kits ELISA específicos para a espécie (EastBiopharm, China) e expressa como ng/mg de proteína tecidual.

### Análises estatísticas

A análise estatística foi realizada com o software SPSS 22 (SPSS Inc., Chicago, IL, EUA). Todos os dados foram apresentados como média ± desvio padrão (DP). Os testes de Shapiro-Wilk ou Levene foram utilizados para determinar a normalidade dos dados ou igualdade das variâncias dos erros. Todos os parâmetros foram analisados estatisticamente por análise de variância (ANOVA) de três vias com tempos de amostragem, tratamento com LC e condição diabética como fatores. Quando a interação e/ou os efeitos principais eram significativos, as médias eram comparadas entre os diferentes grupos experimentais em diferentes momentos utilizando o teste *post hoc* de comparação múltipla de Tukey. Os gráficos foram desenhados utilizando o software Graphpad Prism 8 (GraphPad Software Inc., San Diego, CA, EUA). Não utilizamos métodos estatísticos para predeterminar o tamanho da amostra. Os tamanhos das amostras foram estimados com base na disponibilidade da amostra, questões éticas sobre a realização de objetivos experimentais sem desperdiçar muitos animais e estudos experimentais anteriores.^11 , 16^ Um valor de p < 0,05 foi considerado estatisticamente significante.

## Resultados

**Figura 1:**
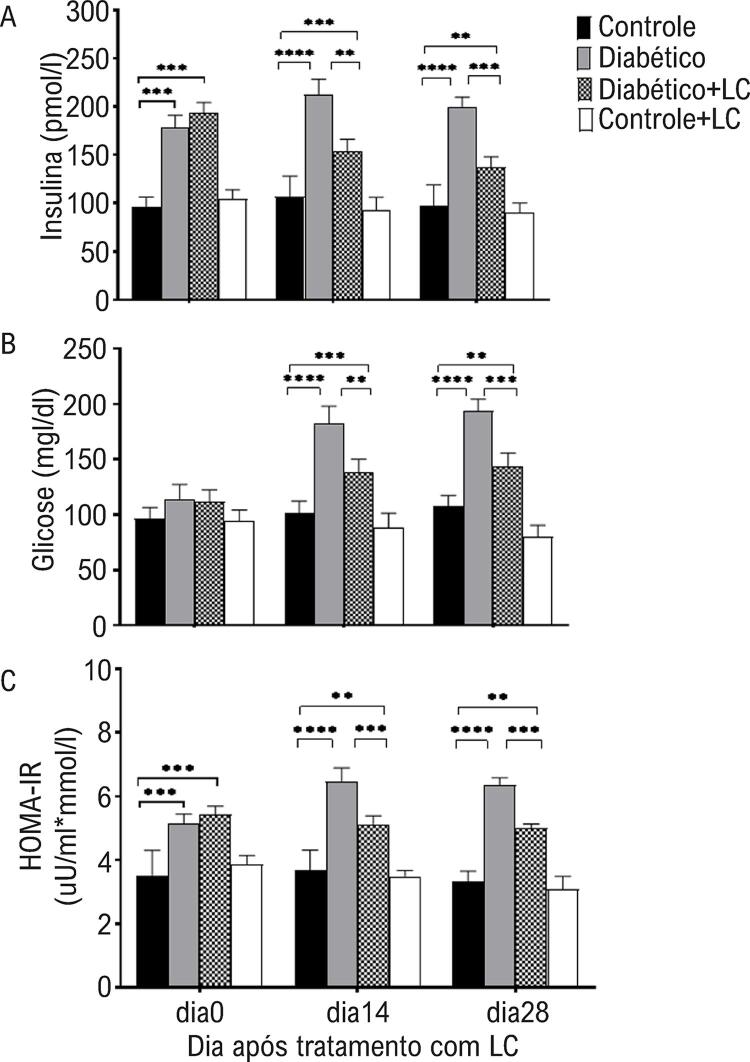
– Níveis séricos de insulina (A), glicose (B) e índice HOMA-IR em diferentes grupos experimentais nos dias 0, 14 e 28 após o tratamento com LC. Os dados são médias ± DP. O nível de significância entre os grupos em cada momento de amostragem foi estabelecido em *p<0,05, **p<0,01, ***p<0,001, ****p<0,0001.

Nossos resultados mostraram que o consumo por 5 semanas de uma dieta hipercalórica concomitante à injeção de baixa dose de STZ induziu um fenótipo de DM2 caracterizado por hiperinsulinemia e hiperglicemia. O efeito dos fatores principais (condição diabética; 2 níveis, tratamento com LC; 2 níveis e tempo de amostragem; 3 níveis) e sua interação em cada variável são mostrados na tabela 1 . Uma análise ANOVA de três vias indicou um efeito principal do tempo de amostragem (p = 0,0031), tratamento com LC (p = 0,0001), condição diabética (p = 0,0001) e interação do tempo de amostragem × tratamento LC × condição diabética (p = 0,0018) no nível de insulina em diferentes grupos experimentais ( Tabela 1 ). As alterações nos níveis de insulina no soro em camundongos diabéticos tratados com LC são mostradas na Figura 1A . O nível sérico de insulina aumentou significativamente no dia 7 após a indução do diabetes (dia 0 do tratamento com LC) em relação aos animais controle saudáveis e permaneceu elevado até o dia 28 do experimento (p<0,05). O nível sérico de insulina diminuiu de forma significante em camundongos diabéticos após duas e quatro semanas de tratamento com LC em comparação com camundongos diabéticos não tratados (p <0,05) ( Figura 1A ). O tratamento de camundongos diabéticos com LC por mais tempo, entre 14 e 28 dias de tratamento, não foi mais eficaz na redução do nível de insulina (p>0,05) ( Figura 1A ).

**Tabela 1:** – Resultados da análise de ANOVA de três vias para determinação dos efeitos da condição diabética (D), tempo de amostragem (TA) e tratamento com LC (LC) e suas interações (D × TA × LC) em cada variável

Variável	Fatores principais						Interações dos fatores principais	

	Condição diabética (D)		Tratamento com LC		Tempos de amostragem (TA)		D×LC×TA	

	p value	F (1,48)	p value	F (1,48)	p value	F (2,48)	p value	F (2,48)
Insulina	p = 0,0001	305,92	p = 0,0001	17,41	p = 0,0031	4,89	p = 0,0018	3,73
Glicose	p = 0,0002	6182,2	p = 0,0005	1248,2	p = 0,0002	634,6	p = 0,0002	67,59
HOMA-IR	p = 0,0005	447,02	p = 0,0017	12,95	p = 0,0019	8,12	p = 0,0015	8,91
MRNA da quemerina adiposa	p = 0,0003	1761,2	p = 0,0003	1040,3	p = 0,0008	125,45	p = 0,0007	319,21
MRNA da quemerina cardíaca	p = 0,0002	5546,3	p = 0,0001	187,6	p = 0,0002	73,36	p = 0,0002	67,9
Proteína quemerina adiposa	p = 0,0002	2267,2	p = 0,0002	762,21	p = 0,0002	70,23	p = 0,0003	64,22
Proteína quemerina cardíaca	p = 0,0003	1651,4	p = 0,0002	641,22	p = 0,0003	62,27	p = 0,0004	64,12
MRNA do CMKLR1 adiposo	p = 0,0001	8376,2	p = 0,0001	306,99	p = 0,0001	96,51	p = 0,0001	65,73
MRNA do CMKLR1 cardíaco	p = 0,0001	2172,3	p = 0,0002	65,04	p = 0,0003	75,59	p = 0,0002	7,37
Quemerina sérica	p = 0,0003	3759,4	p = 0,0005	183,59	p = 0,0003	423,2	p = 0,0003	91,87
IL1-β	p = 0,0005	2042,1	p = 0,0002	53,12	p = 0,0004	375,6	p = 0,0008	68,46
TNF-α	p = 0,0001	4644,3	p = 0,0002	475,6	p = 0,0002	2516,2	p = 0,0002	439,17

Uma análise ANOVA de três vias indicou um efeito principal do tempo de amostragem (p = 0,0002), tratamento com LC (p = 0,0005), condição diabética (p = 0,0002) e interação do tempo de amostragem × tratamento com LC × condição diabética (p = 0,0002) na concentração de glicose sérica em diferentes grupos experimentais. O nível de glicose sérica foi significativamente maior em camundongos diabéticos nos dias 14 e 28 após a indução do diabetes em comparação com animais saudáveis (p<0,05), enquanto a administração de dieta suplementada com HF/HC e injeção de STZ não apresentou efeito significativo no nível de glicose sérica durante a primeira semana de indução experimental do diabetes (p>0,05) ( Figura 1B ). Observou-se que o tratamento de camundongos diabéticos com LC resultou em redução significativa do açúcar no sangue, conforme observado nos dias 14 e 28, em comparação com camundongos diabéticos não tratados (p <0,05) ( Figura 1B ). A ingestão de LC não teve um efeito evidente no nível de glicose sérica em camundongos saudáveis (p>0,05) ( Figura 1B ).

**Figura 2:**
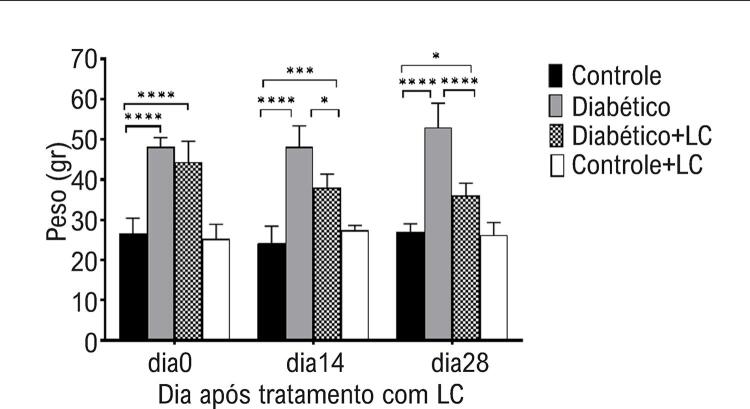
– Mudanças no peso corporal em diferentes grupos experimentais nos dias 0, 14 e 28 após o tratamento com LC. Os dados são médias ± DP. O nível de significância entre os grupos em cada momento de amostragem foi estabelecido em *p <0,05, **p <0,01, ***p <0,001, ****p <0,0001.

Uma análise ANOVA de três vias indicou um efeito principal do tempo de amostragem (p = 0,0019), tratamento com LC (p = 0,0017), condição diabética (p = 0,0005) e interação do tempo de amostragem × tratamento LC × condição diabética (p = 0,0015) no índice HOMA-IR em diferentes grupos experimentais. O índice HOMA-IR em animais diabéticos aumentou significativamente no 14º e 28º dias do período experimental em relação aos camundongos saudáveis (p<0,05), enquanto não apresentou alteração significativa na primeira semana após a indução experimental do diabetes (p >0,05) ( Figura 1C ). A administração oral de LC teve efeito de redução significativa no índice HOMA-IR nos dias 14 e 28 do período experimental em camundongos diabéticos (p <0,05) ( Figura 1C ). Como mostrado na Figura 2 , foi observado maior peso corporal em camundongos que receberam dieta suplementada com HF/HC em todos os momentos do experimento em comparação com camundongos saudáveis (p<0,05). Durante as quatro semanas de observação dos camundongos diabéticos tratados com LC, houve uma perda de peso, em relação ao dia 0, ou seja, antes do início do tratamento (p <0,05) ( Figura 2 ).

**Figura 3:**
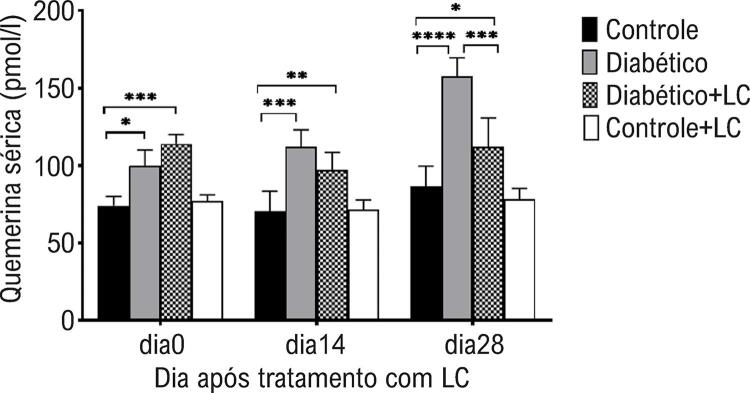
– Níveis séricos de quemerina em diferentes grupos experimentais nos dias 0, 14 e 28 após o tratamento com LC. Os dados são médias ± DP. O nível de significância entre os grupos em cada momento de amostragem foi estabelecido em *p<0,05, **p<0,01, ***p <0,001, ****p <0,0001.

Em relação à concentração de quemerina sérica, uma ANOVA de três vias mostrou um efeito principal do tempo de amostragem (p = 0,0003), tratamento com LC (p = 0,0005), condição diabética (p = 0,0003) e interação do tempo de amostragem × tratamento LC × condição diabética (p = 0,0003). Nossos resultados mostraram que os níveis séricos de quemerina foram significativamente maiores no grupo diabético do que no grupo controle em todos os momentos de amostragem, com o nível mais alto no dia 28 do experimento (p<0,05). Duas semanas após o tratamento com LC, o nível de quemerina sérica de camundongos diabéticos não mostrou diferença significante com o de camundongos diabéticos não tratados (p>0,05), enquanto um efeito de redução significante no nível de quemerina sérica foi observado quando camundongos diabéticos consumiram LC por 28 dias (p< 0,05) ( Figura 3 ).

**Figura 4:**
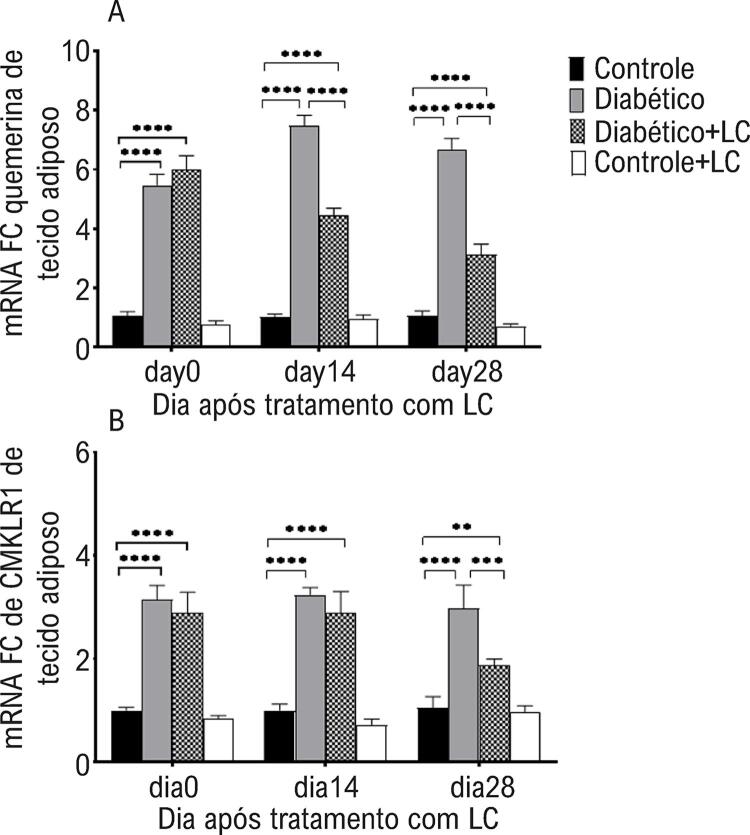
– Níveis de expressão gênica de quemerina (A) e CMKLRI (B) no tecido adiposo de diferentes grupos experimentais nos dias 0, 14 e 28 após o tratamento com LC. O método qRT-PCR foi utilizado para análise da expressão relativa dos genes estudados. GAPDH foi utilizado como gene de housekeeping. O nível de significância entre os grupos em cada momento de amostragem foi estabelecido em *p<0,05, **p<0,01, ***p<0,001, ****p<0,0001.

Em relação aos níveis de quemerina do músculo cardíaco e de mRNA do CMKLR1, uma ANOVA de três vias mostrou um efeito principal do tempo de amostragem (quemerina p = 0,0001, CMKLR1 p = 0,0003), tratamento com LC (quemerina, p = 0,0002; CMKLR1, p = 0,0002), condição diabética (quemerina, p = 0,0002; CMKLR1, p = 0,0001) e interação entre três fatores (quemerina, p<0,0001; CMKLR1, p = 0,0002). Os níveis de mRNA e proteína da quemerina e CMKLRI no tecido cardíaco de camundongos diabéticos foram maiores do que os dos animais saudáveis em todos os momentos de amostragem após a indução do diabetes (p <0,05) ( Figura 4 e Tabela 2 ). O consumo oral de LC por duas semanas não teve efeito significativo na redução da expressão da quemerina ou CMKLRI no tecido cardíaco de camundongos diabéticos (p>0,05), enquanto 28 dias após o tratamento com LC a expressão dos genes estudados foi reduzida no tecido cardíaco de camundongos diabéticos em comparação com os animais diabéticos não tratados (p<0,05) ( Figura 4 , Tabela 2 ).

**Tabela 2:** – Nível de quemerina no tecido cardíaco dos grupos experimentais. Os dados são apresentados como média ± DP. Letras minúsculas diferentes (a, b) demonstram diferenças significativas entre os grupos em cada dia do experimento (p<0,05). Letras maiúsculas diferentes (A, B) demonstram diferenças significativas entre os tempos de tratamento em cada grupo (p <0,05)

Nível de quemerina no tecido cardíaco (ng/mg proteína)			

Dia após o tratamento com LC			

	0	14	28
Controle	2,47 ± 0,42^aA^	2,56 ± 0,27^aA^	2,27 ± 0,22^aA^
Diabético	3,01 ± 0,31^aA^	4,97 ± 0,48^bB^	5,51 ± 0,41^bB^
Diabético + LC	2,81 ± 0,16^aA^	4,42 ± 0,38^bB^	3,13 ± 0,29^cA^
Controle + LC	2,32 ± 0,38^aA^	2,67 ± 0,33^aA^	2,37 ± 0,28^aA^

*LC: l-carnitina.*

**Figura 5:**
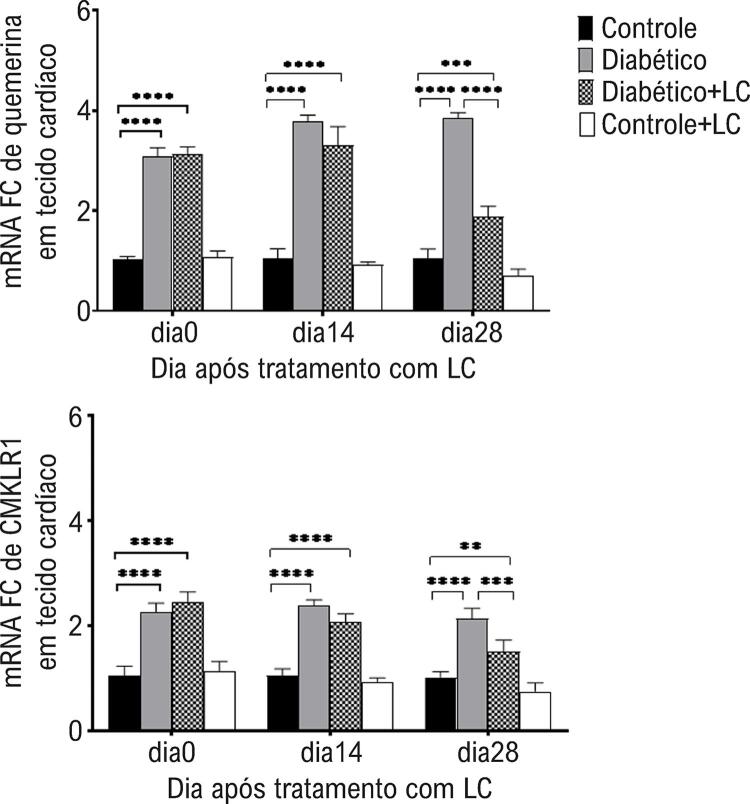
– Níveis de expressão gênica de quemerina (A) e CMKLRI (B) no tecido cardíaco de diferentes grupos experimentais nos dias 0, 14 e 28 após o tratamento com LC. O método qRT-PCR foi utilizado para análise da expressão relativa dos genes estudados. GAPDH foi utilizado como gene de housekeeping. Os dados são médias ± DP. O nível de significância entre os grupos em cada momento de amostragem foi estabelecido em *p<0,05, **p<0,01, ***p<0,001, ****p<0,0001.

Em relação à quemerina adiposa e níveis de mRNA e proteína de CMKLR1, uma ANOVA de três vias mostrou um efeito principal do tempo de amostragem (quemerina, p = 0,0008; CMKLR1 p = 0,0001), tratamento com LC (quemerina, p = 0,0003; CMKLR1, p = 0,0001), condição diabética (quemerina, p = 0,0003; CMKLR1 p = 0,0001) e interação entre os três fatores (quemerina, p = 0,0007; CMKLR1, p = 0,0001). Observou-se que os animais que receberam dieta suplementada com HF/HC apresentaram níveis maiores de proteína e de mRNA de quemerina e CMKLRI no tecido adiposo no 7º, 14º e 28º dias após a alimentação final em comparação com os animais que receberam dieta normal (p <0,05) ( Figura 5 A-B , Tabela 3 ). O tratamento de camundongos diabéticos com LC por 14 ou 28 dias resultou em uma redução significativa dos níveis de proteína quemerina e mRNA no tecido adiposo de animais diabéticos quando comparados com camundongos diabéticos não tratados (p<0,05) ( Figura 5A , Tabela 2 ). Os animais que receberam LC por 14 dias não apresentaram alteração significativa na expressão de CMKLRI (p >0,05), enquanto aqueles que foram tratados com LC por 28 dias apresentaram menor expressão de CMKLRI quando comparados com camundongos diabéticos não tratados (p<0,05) ( Figura 5B ).

**Tabela 3:** – Nível de quemerina no tecido adiposo de diferentes grupos experimentais. Os dados são apresentados como média ± DP. Letras minúsculas diferentes (a, b) demonstram diferenças significativas entre os grupos em cada dia do experimento (p<0,05). Letras maiúsculas diferentes (A, B) demonstram diferenças significativas entre os tempos de tratamento em cada grupo (p <0,05)

Nível de quemerina no tecido adiposo (ng/mg proteína)			

Dia após o tratamento com LC			

	0	14	28
Controle	7,62 ± 2,58^aA^	8,11 ± 1,32^aA^	6,49 ± 1,07^aA^
Diabético	13,22 ± 3,61^bA^	14,78 ± 2,59^bA^	16,72 ± 97^bB^
Diabético + LC	12,64 ± 2,09^bA^	10,36 ± 1,78^cB^	10,97 ± 3,12^bB^
Controle + LC	6,82 ± 1,94^aA^	7,09 ± 2,06^aA^	7,44 ± 1,19^aA^

*LC: l-carnitina.*

Em relação à concentração sérica de IL1-β e TNF-α, uma ANOVA de três vias mostrou um efeito principal do tempo de amostragem (IL1-β, p = 0,0004 e TNF-α, p = 0,0002), tratamento (IL1-β, p = 0,0002 e TNF-α, p = 0,0002), condição diabética (IL1-β; p = 0,0005, TNF-α p = 0,0001) e interação entre três fatores (IL1-β, p = 0,0008; TNF-α, p = 0,0002). Os níveis séricos de IL1-β e TNF-α aumentaram significativamente no grupo diabético em comparação com o grupo controle nos dias 14 e 28 após a indução do diabetes (p<0,05) ( Tabelas 4 e 5 ). O tratamento de camundongos diabéticos com LC por 14 dias não teve efeitos significativos nos níveis séricos de IL-1β e TNF-α em comparação com camundongos diabéticos não tratados (p>0,05). No entanto, quatro semanas após o tratamento com LC, as concentrações séricas de ambos os fatores inflamatórios foram significativamente reduzidas em comparação com camundongos diabéticos não tratados.

**Tabela 4:** – Nível sérico de IL1-β em diferentes grupos experimentais. Os dados são apresentados como média ± DP. Letras minúsculas diferentes (a, b) demonstram diferenças significativas entre os grupos em cada dia do experimento (p<0,05). Letras maiúsculas diferentes (A, B) demonstram diferenças significativas entre os tempos de tratamento em cada grupo (p<0,05). LC: l-carnitina

Nível sérico de TNF-α (pmol/L)			

Dia após o tratamento com LC			

	0	14	28
Controle	0,71 ± 0,19^aA^	0,65 ± 0,10^aA^	0,75 ± 0,09^aA^
Diabético	0,65 ± 0,11^aA^	3,92 ± 0,38^bB^	7,10 ± 0,68^bC^
Diabético + LC	1,33 ± 0,16^bA^	3,34 ± 0,50^bB^	3,94 ± 0,43^cB^
Controle + LC	0,78 ± 0,18^aA^	0,98 ± 0,16^aA^	0,57 ± 0,11^dB^

*LC: l-carnitina.*

**Tabela 5:** – Nível sérico de TNF-α em diferentes grupos experimentais nos dias 0, 14 e 28 após o tratamento com LC. Os dados são apresentados como média ± DP. Letras minúsculas diferentes (a, b) demonstram diferenças significativas entre os grupos em cada dia do experimento (p<0,05). Letras maiúsculas diferentes (A, B) demonstram diferenças significativas entre os tempos de tratamento em cada grupo (p<0,05)

Nível sérico de IL1-β (pmol/L)			

Dia após o tratamento com LC			

	0	14	28
Controle	0,93 ± 0,11^aA^	1,02 ± 0,14^aA^	0,89 ± 0,31^aA^
Diabético	1,23 ± 0,33^aA^	2,96 ± 0,12^bB^	5,78 ± 0,21^bB^
Diabético + LC	1,33 ± 0,26^aA^	2,23 ± 0,27^bB^	3,46 ± 0,12^cC^
Controle + LC	1,07 ± 0,24^aA^	0,98 ± 0,19^aA^	0,82 ± 0,41^aA^

*LC: l-carnitina.*

## Discussão

No presente estudo, foi estudado o efeito da suplementação de LC na expressão de quemerina e seu receptor nos tecidos adiposo e cardíaco de ratos obesos e resistentes à insulina experimentalmente induzidos. De acordo com estudos anteriores em modelo de roedor de diabetes tipo 2, nossos resultados mostraram que o consumo de cinco semanas de dieta suplementada com HF/HC, juntamente com a injeção de uma única dose baixa de STZ em camundongos, resultou em resistência à insulina e obesidade, caracterizada por hiperglicemia, hiperinsulinemia e aumento do índice HOMA-IR e peso corporal.^16 , 18 , 19^ Foi observado que a suplementação da dieta com HF/HC por cinco semanas resultou em hiperinsulinemia sem alteração óbvia da glicose sanguínea, enquanto após duas semanas de alimentação com dieta hipercalórica, os animais mostraram sintomas séricos de resistência à insulina, incluindo hiperinsulinemia e hiperglicemia. Esses achados sugerem que as alterações metabólicas séricas induzidas por esse tratamento foram mais semelhantes às do diabetes tipo 2 do que às do diabetes tipo 1.

Nossos resultados mostraram que a expressão de quemerina e seu receptor CMKLRI nos tecidos adiposo e cardíaco de camundongos diabéticos, aumentou após a indução do diabetes experimental. Também foi observado que animais diabéticos apresentaram maior concentração sérica de quemerina em comparação com animais saudáveis. Esse achado sugere que mudanças simultâneas na quantidade de quemerina e seu receptor no estado diabético exacerba a função desse hormônio nos tecidos-alvo e isso pode desempenhar um papel importante no desenvolvimento de distúrbios funcionais dos tecidos adiposo e cardíaco no estado diabético. A maior parte dos dados humanos e de animais suporta uma ligação entre quemerina, obesidade e síndrome metabólica, um grupo de distúrbios metabólicos que aumentam o risco de DCV. De acordo com nossos resultados, estudos em humanos e animais relataram os achados paralelos de que animais obesos e diabéticos têm níveis circulantes elevados de quemerina em comparação com indivíduos ou animais saudáveis ou magros.^22 , 23^ Vários mecanismos possíveis podem ser aceitos sobre os efeitos do aumento da expressão de quemerina e CMKLRI no início e progressão da resistência à insulina em animais diabéticos. Sabe-se agora que a inflamação tem um papel importante na resistência à insulina do tecido adiposo e na disfunção do músculo cardíaco.^24^ A obesidade está correlacionada a um aumento significativo na produção de citocinas pró-inflamatórias, como IL-1β e TNF-α, que podem induzir resistência à insulina e morte celular no músculo cardíaco.^25 , 26^ Nossos achados mostraram níveis séricos aumentados de IL1-β e TNF-α após a indução do diabetes. Os níveis séricos de quemerina se correlacionam com os níveis de citocinas pró-inflamatórias, tais como TNF-α, IL1-β e IL-6.^6 , 7 , 25^ A quemerina também tem efeitos adversos sobre os cardiomiócitos através da indução da caspase 9 e apoptose celular mediada por AKT.^10^ Tomados em conjunto, esses dados permitem a conclusão de que a superexpressão observada de quemerina e seu receptor em tecidos adiposos e cardíacos de camundongos diabéticos pode ter um papel importante na progressão da resistência à insulina e disfunções cardíacas no estado diabético.

Nossos resultados mostraram que a administração de LC por 4 semanas pode atenuar a expressão da quemerina e de seu receptor nos tecidos adiposo e cardíaco de camundongos diabéticos. Essas alterações foram associadas à melhora dos sintomas de resistência à insulina e à redução dos níveis séricos de quemerina e de alguns fatores inflamatórios. Em concordância com os resultados do presente estudo, estudos anteriores mostraram que o uso de LC leva ao aumento da sensibilidade geral à insulina e da captação de glicose mediada pela insulina no corpo inteiro em animais ou humanos com diabetes tipo 2.^16 , 26^ Evidências consideráveis apoiam que o suprimento excessivo de ácidos graxos e a obesidade levam ao acúmulo ectópico de vários metabólitos lipídicos no músculo cardíaco e possivelmente em outros tecidos que são prejudiciais à sinalização da insulina, levando à resistência insulínica.^27^ As células do músculo cardíaco não podem sintetizar LC *de novo* e devem obter a LC de forma exógena através da carnitina/transportador de cátions orgânicos 2.^28^ Com base nesses achados, sugerimos que a suplementação de LC na dieta pode restaurar o *pool* de LC e reduzir os metabólitos lipídicos e a lipotoxicidade nos tecidos cardíaco e adiposo.

Com base em nosso conhecimento, os efeitos da LC no nível sistêmico de quemerina ou sua expressão no tecido cardíaco de animais ou humanos com diabetes não foram investigados até o momento. Nossos resultados, pela primeira vez, mostraram que os níveis de proteína e mRNA da quemerina e seu receptor eram reduzidos no tecido cardíaco de camundongos diabéticos, concomitantemente ao aumento da sensibilidade à insulina em todo o corpo. Achados anteriores mostraram que os níveis de mRNA e proteína da quemerina estavam elevados no tecido adiposo epicárdico de pacientes com doença arterial coronariana.^29^ O nível sérico de quemerina também está elevado em pacientes com doença arterial coronariana e correlacionado com a gravidade e extensão da estenose coronariana e vários parâmetros cardiometabólicos.^30^ Foi observado que a quemerina aumenta a apoptose e a atividade da caspase 9 em cardiomiócitos murinos e melhora os parâmetros cardioprotetores no coração de rato perfundido isolado.^10 , 31^ O tratamento com quemerina também altera o destino das células mioblásticas, da miogênese para a adipogênese, que é caracterizada pelo aumento dos níveis de ERO e do conteúdo de TG das células tratadas.^32^ Tomados em conjunto, concluiu-se que a redução da expressão da quemerina e de seu receptor no tecido cardíaco de camundongos diabéticos após o tratamento com LC pode atenuar o efeito adverso da quemerina nos cardiomiócitos em animais diabéticos. Mais estudos são necessários para confirmar esta opinião.

Embora os resultados do presente estudo tenham mostrado que o tratamento com LC pode atenuar o aumento da expressão da quemerina nos tecidos-alvo, os mecanismos moleculares que regulam sua expressão permanecem pouco compreendidos. Nossos resultados mostraram que o tratamento com LC pode reduzir os níveis séricos de TNF-α e IL1-β em ratos obesos diabéticos. Foi observado que essas citocinas pró-inflamatórias induzem a expressão e secreção de mRNA da quemerina pelos adipócitos 3T3-L1.^33^ O nível sérico de quemerina também está associado ao nível sérico de TNF-α em pacientes obesos.^34^ Com base nesses achados, a redução da expressão de quemerina em camundongos diabéticos pode ser devido a uma diminuição na secreção de mediadores inflamatórios. A melhora da hiperinsulinemia também pode ser outro mecanismo da LC na redução da expressão da quemerina. Nossos resultados mostraram que a LC pode atenuar o aumento do nível de insulina em camundongos obesos e diabéticos. Como a insulina pode aumentar a secreção de quemerina do tecido adiposo *in vitro* e em explantes de tecido,^35^ sugerimos que a redução da insulina em animais tratados com LC pode ter um papel regulador na expressão da quemerina nos tecidos adiposo e cardíaco de camundongos diabéticos.

Esse estudo tem algumas limitações. Em nosso estudo, a expressão gênica foi medida no tecido cardíaco total em animais experimentais. Tendo em vista que vários tipos de células cardíacas, como cardiomiócitos e fibroblastos cardíacos, podem ter diferentes ações na patogênese da doença cardíaca, nossos dados não permitiram distinguir perfis de expressão entre os vários tipos de células. Além disso, foram confirmadas diferenças nos perfis de expressão gênica entre os tecidos cardíacos dos ventrículos direito e esquerdo e nos átrios, além das diferenças entre os tipos de células cardíacas. Assim, não diferenciamos a expressão gênica entre diferentes partes do tecido cardíaco nos animais experimentais. Outra limitação está relacionada ao fato de que medimos todos os fatores em animais adultos jovens e em um modelo experimental de diabetes de curto prazo. A este respeito, é interessante notar que o envelhecimento e o diabetes de longa duração são, de longe, o fator de risco dominante para o desenvolvimento de doenças cardiovasculares, e a prevalência de doenças cardiovasculares aumenta dramaticamente com o aumento da idade. Não determinamos se o tratamento com LC em ratos idosos ou diabetes de longa duração leva a uma downregulação semelhante à expressão de quemerina e seu receptor no tecido cardíaco de ratos diabéticos.

## Conclusão

Em resumo, nossos dados mostraram que o consumo de uma dieta hipercalórica resultou em obesidade, resistência à insulina e upregulação da quemerina e seu receptor, CMKLRI, nos tecidos adiposo e cardíaco de camundongos. O tratamento de camundongos diabéticos com LC pode atenuar os sintomas de resistência à insulina e suprimir a superexpressão de quemerina em animais diabéticos. Os resultados do presente experimento ajudam a compreender o novo efeito regulatório da LC na expressão do gene cardíaco em condições de obesidade e diabetes. Mais estudos são necessários para fornecer mais evidências sobre a terapia nutricional com LC para o manejo não farmacológico de pacientes com diabetes e DCV com base na regulação dos componentes da quemerina no músculo cardíaco.
