# Supplementary material for: Novo Efeito Cardioprotetor da L-Carnitina em Camundongos Obesos Diabéticos: Regulação da Expressão de Quemerina e CMKLRI no Coração e Tecidos Adiposos
Source: Arq Bras Cardiol. 2021 Oct 6;117(4):715–25. [Article in Portuguese] doi: 10.36660/abc.20200044 (PMC8528366; doi:10.36660/abc.20200044)
Supplement: Translation

# Translation: Novel Cardioprotective Effect of L-Carnitine on Obese Diabetic Mice: Regulation of Chemerin and CMKLRI Expression in Heart and Adipose Tissues

**Authors:** Rezvan Amiri, Mohammad Reza Tabandeh, Seyed Ahmad Hosseini

**Affiliations:** 1 Iran Department of Basic Sciences, Division of Biochemistry and Molecular Biology, Faculty of Veterinary Medicine, Shahid Chamran University of Ahvaz, Ahvaz – Iran; 2 Iran Department of Nutrition Science, Ahvaz Jundishapur University of Medical Sciences, Ahvaz – Iran

**Keywords:** Diabetes Mellitus, Carnitine, Chemokines

## Abstract

**Background:**

L-carnitine (LC) has many beneficial effects on diabetic animals and humans, but its regulatory effect on chemerin as an inflammatory cytokine, and its receptor in diabetes status is unknown.

**Objectives:**

The present study aimed to investigate the regulatory effect of LC on the expression of chemerin and chemokine-like receptor I (CMKLRI) in adipose and cardiac tissues of diabetic mice.

**Methods:**

Sixty NMARI mice were divided into four groups including control, diabetic, diabetic + LC supplementation and control + LC supplementation. Diabetes was induced by feeding the animals a high-calorie diet for 5 weeks and injection of Streptozotocin. The animals were treated with 300 mg/kg LC for 28 days. On days 7, 14, and 28 after treatment, the mRNA and protein levels of chemerin and CMKLRI in the cardiac and adipose tissues of the animals were determined using qPCR analysis and ELISA. Insulin resistance indices were also measured in all experimental groups. Differences with p <0.05 were considered significant.

**Results:**

Chemerin and CMKLRI expressions levels were increased in cardiac and adipose tissues of diabetic mice on days 14 and 28 after diabetes induction, concurrent with the incidence of insulin resistance and increased levels of circulating chemerin (p<0.05). The treatment with LC caused a significant decrease in the expression of both genes in studied tissues and the reduction of insulin resistance symptoms and serum chemerin levels (p<0.05).

**Conclusion:**

The results suggest that LC treatment were able to downregulate the expression of chemerin and CKLR1 in cardiac and adipose tissues of obese, diabetic experimental animals.

## Introduction

A growing body of evidence has shown the existence of a complex and multifaceted biochemical and molecular relationship between diabetes mellitus (DM), obesity and cardiovascular diseases (CVDs).^1^ One possible mechanism linking DM and obesity with subsequent CVDs is low-grade inflammation in adipose tissue.^2^ The adipose tissue of animals or humans with DM and insulin resistance secretes a variety of cytokines or adipocytokines. The abnormal secretion of these adipocytokines contributes to increased inflammation and lipid accumulation and can lead to the development of endothelial and myocardial dysfunction and cardiomyopathies.^2 , 3^ The alterations in the expression or secretion of pro-inflammatory and anti-inflammatory adipokines in cardiomyocytes and adipose tissue have been implicated in the pathogenesis of metabolic syndrome and CVDs in obese animals or humans.^2^

Chemerin is a recently identified adipokine that acts as a chemoattractant protein and mediates its effects through a G-protein coupled receptor, the chemokine-like receptor 1 (CMKLRI), also known as ChemR23.^4 - 7^ Previous studies demonstrated that chemerin had a role in various cardiovascular pathologies, including the development of hypertension, progression of atherosclerotic lesions, and impaired heart function in patients with dilated cardiomyopathy.^8^ Chemerin can promote endothelial inflammation, and induce apoptosis in murine cardiomyocytes, demonstrating the potential role of chemerin in cardiomyocyte dysfunctions.^9 , 10^

Recently, mitochondrial-targeting nutrients, such as lipoic acid, L-carnitine (LC), nicotinamide and biotin have been given more attention regarding the improvement of diabetes-related inflammatory and cardiovascular complications.^11 , 12^ L-carnitine (LC) is a conditionally essential nutrient that facilitates fatty acid transport to the oxidation sites in the mitochondria.^13 , 14^

There is increasing evidence that LC supplementation may be beneficial in treating insulin-resistance and obesity-related metabolic disturbances in diabetic patients.^11^ Various studies have shown that LC has beneficial effects on the prevention and improvement of cardiovascular disease, including chronic heart failure, anterior acute myocardial infarction, coronary artery disease, myocardial remodeling and atrial fibrillation.^12 , 14^ Several mechanisms have been reported regarding the protective role of LC in cardiac cells under hyperglycemic conditions, including improvement of cardiac energy homeostasis, oxidative stress attenuation and hypoxic cell damage and apoptosis reduction.^15 , 16^ Limited evidence has also shown that LC may affect serum chemerin levels in obese children.^17^ Based on our knowledge, a few studies have focused on the role of LC supplementation on chemerin expression regulation, particularly in cardiac and adipose tissues of diabetic animals. The aim of the present study was to investigate the effects of LC supplementation on chemerin and CMKLRI expression in adipose and cardiac tissues in an experimental model of obesity and diabetes induced by a high-calorie diet fed to mice.

## Materials and methods

### Animals

Sixty male NMARI mice (25±2 g) were selected from the Center of Laboratory Animals of the School of Veterinary Medicine of Shahid Chamran University of Ahvaz, Iran. All the mice were kept under controlled environmental conditions, with an average temperature of 23 ± 1 °C, a 12:12 h light/darkness cycle and free access to water and special diet for mice prepared as pellet (Pars, Tehran, Iran). All experimental protocols were approved by the Ethics Committee for Research in Animals and Humans of Shahid Chamran University of Ahvaz (EE/98.24.3.26209/SCU.AC.IR). All animal experimentations were carried out in accordance with the guidelines for the care and use of laboratory animals (NIH publication n. 86-23). There was an adaptation period of 7 days before the start of the experiment.

### Experimental design

The animals (n=60) were randomly divided into four equal groups. Randomization was achieved by allocating the animals of the same sex, age and weight in different experimental groups using random number tables. Two groups were fed a high-energy diet, which was prepared by adding 20% sucrose (w/w) and 10% lard (w/w) into the diets for 5 weeks and described as HF/HC (n=30), whereas the other ones continued to consume normal diets for the same period, serving as a control groups (n=30). After 5-weeks receiving the HF/HC diets, the diabetic groups were treated with a single intraperitoneal injection of STZ (Sigma, Germany) (30 mg/kg body weight) prepared in a citrate buffer solution.^18 , 19^

Blood glucose was measured five days after STZ treatment using a hand-held glucometer (Medisign, China) and the presence of diabetes was confirmed if serum glucose was over 6.5 mmol/L and if the HOMA–IR was high in comparison to the control group.^20^ The day following the confirmation of diabetes was considered as day 0 of LC treatment. One diabetic group was treated with LC at a dose of 300 mg/kg (n=15) in drinking water, while concomitantly being fed the HF/HC diets for 28 days, while the other diabetic group (n=15) (diabetic control) were fed only HF/HC diets for the same period.^18 , 19^ One control group (n=15) that received a normal diet and the other control group (n=15) (LC-treated control) that consumed LC at dose of 300 mg/kg in drinking water for 28 days were also considered for the analyses.

### Sampling

Animal euthanasia was carried out with a combination of ketamine (100 mg/kg) and xylazine (10 mg/kg) respectively, on days 0, 14 and 28 of LC treatment. Blood samples were collected, and sera were separated and stored at -20°C for later use. Hearts and visceral adipose tissues were separated and kept at –70°C until use.

### Biochemical parameter measurements

Serum insulin and chemerin levels were determined using the species-specific ELISA kits (EastBiopharm, China). Serum glucose was measured by commercial kits (PishtazTeb, Iran) according to the manufacturer’s recommendations. Serum IL1-β and TNF-α levels were determined using species-specific ELISA kits, as recommended by the manufacturer (Biovision Inc. USA)

### HOMA–IR Estimation

The HOMA-IR factor was calculated using the following formula:

HOMA index = Fasting insulin (µU/ml) × Fasting glucose (mmol/L) /22.5. The rising and falling of HOMA–IR values in relation to control, healthy animals indicate increase and decrease in insulin sensitivity, respectively.^20^

### RNA isolation and cDNA synthesis

Total RNA was extracted from 100 mg of heart and adipose tissues using RNX^TM^isolation reagent according to the manufacturer’s procedure (SinaClon, Tehran, Iran). The samples were treated with DNase I enzyme to avoid DNA contamination. The purity of RNA at a 260/280 OD ratio and RNA integrity were evaluated using an Eppendorf µCuvette G1.0 microvolume measuring cell (Eppendorf BioPhotometer D30, Eppendorf, Germany). Reverse transcription was performed with the YTA cDNA synthesis kit (Yekta tajhiz, Iran) and Eppendorf Thermal Cycler (Germany) using 1 µg of RNA and random hexamer, as recommended by the manufacturer.

### Real-time quantitative RT-PCR

Real-time PCR was performed using the Roche Light-Cycler detection system (Basel, Switzerland) with the qPCR^TM^Green Master Kit for SYBR Green I^®^(Yektatajhiz, Iran). The relative expression level of the chemerin and CMKLRI transcripts were compared to mice GAPDH as the housekeeping gene. Specific sets of primers (Pishgam BioTech, Co, Tehran, Iran) designed for this study were: chemerin (GenBank:NC-007299): 5’- TCTTCACCTACGACCAGTATCAG -3’ and 5’- ACATTATCTGCATAGACCCCATTG -3’ and *CMKLRI* (GenBank:NM-008153.3): 5’- GTACGACGCTTACAACGACT -3’ and 5’- GCACACCAAGCTGTAGATCA -3’, *GAPDH* (GenBank:NM-001034055): 5’- CTCATCTACCTCTCCATCGTCTG -3’ and 5’- CCTGCTCTTGTCTGCCGGTGCTTG -3’ The PCR protocol used consisted of a 5 min denaturation at 94°C followed by 45 cycles at 94^o^C for 15 sec, 60^o^C for 30 sec. Two separate reactions without cDNA or with RNA were performed in parallel as controls. The relative quantification was performed according to the comparative 2^-^method and using Lightcycler 96^®^software. Assay validation to check whether the primer for chemerin and CMKLRI and GAPDH had similar amplification efficiencies was performed as previously described. All qPCR analyses were performed according to The Minimum Information for Publication of Quantitative Real-Time PCR Experiments (MIQE) guideline.^21^

### Determination of tissue chemerin protein

Cardiac and adipose tissues were homogenized in 500 μl RIPA lysis buffer (NaCl; 150 mM, SDS 0.1%, Tris; 25 mM, pH 7.4, NaF; 1 mM, Phenylmethylsulfonyl fluoride 1 mM) with a homogenizer (Heidolph, Schwabach, Germany). The homogenate was centrifuged at 10000×RPM for 15 min at 4°C (Centrifuge 5415 R; Eppendorf AG, Hamburg, Germany). The supernatant was collected and stored at -70°C for subsequent analysis. Protein concentration in the supernatant was estimated using the Bradford method. Chemerin concentration was determined using the species-specific ELISA kits (East Biopharm, China) and expressed as ng/mg of tissue protein.

### Statistical analyses

Statistical analysis was conducted using the SPSS 22 software (SPSS Inc., Chicago, IL, USA). All data were presented as mean ± standard deviation (SD). The Shapiro–Wilk *or Levene's test* was used to *determine* the normality of data or equality of *error variances* . All parameters were statistically analyzed by three-way analysis of variance (ANOVA) with sampling times, LC treatment and diabetic condition as factors. When the interaction and/or the main effects were significant, means were compared between different experimental groups at different time points using Tukey’s multiple-comparison post-hoc test. Graphs were drawn using Graphpad Prism 8 (GraphPad Software Inc., San Diego, CA, USA). We did not use any statistical methods to predetermine sample size. Samples sizes were estimated based on sample availability, ethical issues about accomplishing experimental aims without having to use numerous animals and previous experimental studies.^11 , 16^ A *p* -value below 0.05 was considered statistically significant.

## Results

Our results showed that consumption of a 5-week high calorie diet, concurrent with low-dose injection of STZ induced a T2DM phenotype that was characterized by hyperinsulinemia and hyperglycemia. The effect of the main factors (Diabetic condition, 2 levels; LC treatment, 2 levels; and sampling time, 3 levels) and their interaction with each variable are shown in Table 1 . A three-way ANOVA analysis indicated a main effect of sampling time (p = 0.0031), LC treatment (p = 0.0001), diabetic condition (p = 0.0001) and the interaction of sampling time ×LC treatment × diabetic condition (p = 0.0018) on insulin levels in different experimental groups ( Table 1 ). Changes in serum insulin levels in diabetic mice treated with LC are shown in Figure 1A . Serum levels of insulin were significantly increased at day 7 after diabetes induction (day 0 of LC treatment) in relation to healthy, control animals and it remained high until day 28 of the experiment (p<0.05). Serum insulin levels were significantly decreased in diabetic mice after two and four weeks of treatment with LC, when compared to untreated diabetic mice (p<0.05) ( Figure 1A ). Treatment of diabetic mice with LC for a longer period, between 14 and 28 days of treatment, was no more effective in reducing insulin levels (p>0.05) ( Figure 1A ).

**Table 1:** – Results of three-way ANOVA analysis to determine the effects of the diabetic condition (D), Sampling time (ST) and LC treatment (LC) and their interactions (D × ST × LC) on each variable.

Variable	Main factors						Interactions of main factors	

	Diabetic condition (D)		LC treatment (LC)		Sampling times (ST)		D×LC×ST	

	p value	F (1.48)	p value	F (1.48)	p value	F (2.48)	p value	F (2.48)
Insulin	p = 0.0001	305.92	p = 0.0001	17.41	p = 0.0031	4.89	p = 0.0018	3.73
Glucose	p = 0.0002	6182.2	p = 0.0005	1248.2	p = 0.0002	634.6	p = 0.0002	67.59
HOMA-IR	p = 0.0005	447.02	p = 0.0017	12.95	p = 0.0019	8.12	p = 0.0015	8.91
Adipose chemerin mRNA	p = 0.0003	1761.2	p = 0.0003	1040.3	p = 0.0008	125.45	p = 0.0007	319.21
Cardiac chemerin mRNA	p = 0.0002	5546.3	p = 0.0001	187.6	p = 0.0002	73.36	p = 0.0002	67.9
Adipose chemerin protein	p = 0.0002	2267.2	p = 0.0002	762.21	p = 0.0002	70.23	p = 0.0003	64.22
Cardiac chemerin protein	p = 0.0003	1651.4	p = 0.0002	641.22	p = 0.0003	62.27	p = 0.0004	64.12
Adipose CMKLR1 mRNA	p = 0.0001	8376.2	p = 0.0001	306.99	p = 0.0001	96.51	p = 0.0001	65.73
Cardiac CMKLR1 mRNA	p = 0.0001	2172.3	p = 0.0002	65.04	p = 0.0003	75.59	p = 0.0002	7.37
Serum chemerin	p = 0.0003	3759.4	p = 0.0005	183.59	p = 0.0003	423.2	p = 0.0003	91.87
IL1-β	p = 0.0005	2042.1	p = 0.0002	53.12	p = 0.0004	375.6	p = 0.0008	68.46
TNF-α	p = 0.0001	4644.3	p = 0.0002	475.6	p = 0.0002	2516.2	p = 0.0002	439.17

**Figure 1:** – Serum levels of insulin (A), Glucose (B) and HOMA-IR in different experimental groups on days 0, 14 and 28 of LC treatment. Data are shown as means ± SD. The level of significance between groups at each sampling time was set at *p< 0.05, **p< 0.01, *** p< 0.001, **** p< 0.0001.

A three-way ANOVA analysis indicated a main effect of sampling time *(* p = 0.0002), LC treatment (p = 0.0005), diabetic condition (p = 0.0002) and interaction of sampling time × LC treatment × diabetic condition (p = 0.0002) on serum glucose levels in different experimental groups. The serum glucose levels were found to be significantly higher in diabetic mice on days 14 and 28 after diabetes induction compared with healthy animals (p<0.05), while administration of HF/HC supplemented diet and STZ injection had no significant effect on serum glucose levels during the first one week of experimental induction of diabetes (p>0.05) ( Figure 1B ). It was observed that the treatment of *diabetic mice with LC* resulted in a significant reduction of blood sugar levels, as observed on days 14 and 28 when compared with diabetic, untreated mice (p<0.05) ( Figure 1B ). LC intake had no obvious effect on serum glucose levels in healthy mice (p>0.05) ( Figure 1B ).

A three-way ANOVA analysis indicated a main effect of sampling time *(* p = 0.0019), LC treatment (p = 0.0017), diabetic condition (p = 0.0005) and interaction of sampling time × LC treatment × diabetic condition *(* p = 0.0015) on HOMA-IR in different experimental groups. HOMA-IR in diabetic animals was found to significantly increase on days 14 and 28 of the experimental period in relation to healthy mice (p<0.05), while it showed no significant change in the first week after the experimental induction of diabetes (p>0.05) ( Figure 1C ). Oral administration of LC had a significant lowering effect on HOMA-IR on days 14 and 28 of the experimental period in diabetic mice (p<0.05) ( Figure 1C ). As shown in Fig. 2 , higher body weight was observed in mice that received HF/HC supplemented diet at all times of the experiment, when compared with healthy mice (p<0.05). During the four weeks of observation of the LC-treated diabetic mice, the latter showed weight loss, relative to day 0, i.e., before the start of the treatment (p<0.05) ( Figure 2 ).

**Figure 2:** – Changes in body weight in different experimental groups on days 0, 14 and 28 of LC treatment. Data are means ± SD. The level of significance between groups at each sampling time was set at *p< 0.05, **p< 0.01, *** p< 0.001, **** p< 0.0001.

Regarding serum chemerin levels, a three-way ANOVA analysis showed a main effect of sampling time (p = 0.0003), LC treatment *(* p = 0.0005), diabetic condition (p = 0.0003) and interaction of sampling time × LC treatment × diabetic condition (p = 0.0003). Our results showed that serum chemerin levels were significantly higher in the diabetic group than in the control group at all sampling times, with the highest level being observed on day 28 of the experiment (p<0.05). After two weeks of LC treatment, serum chemerin levels of diabetic mice showed no significant difference with those of untreated diabetic mice (p > 0.05), while a significant lowering effect on serum chemerin levels was observed when diabetic mice consumed LC for 28 days (p<0.05) ( Figure 3 ).

**Figure 3:** – Serum levels of chemerin in different experimental groups on days 0, 14 and 28 of LC treatment. Data are shown as means ± SD. The level of significance between groups at each sampling time was set at *p< 0.05, **p< 0.01, *** p< 0.001, **** p< 0.0001.

As for the chemerin and CMKLR1 mRNA levels in cardiac muscle, a three-way ANOVA analysis showed a main effect of sampling time (chemerin p = 0.0001, CMKLR1 p = 0.0003), LC treatment (chemerin; p = 0.0002, CMKLR1 p = 0.0002), diabetic condition (chemerin; p = 0.0002, CMKLR1 p = 0.0001) and interaction between the three factors (chemerin; p < 0.0001, CMKLR1 p = 0.0002). The mRNA and protein levels of chemerin and CMKLRI in cardiac tissue of diabetic mice were higher than that in normal animals at all sampling times after diabetes induction (p<0.05) ( Figure 4 and Table 2 ). Oral consumption of LC for two weeks had no significant effect on the reduction of chemerin or CMKLRI expression in cardiac tissue of diabetic mice (p>0.05), while the expression of the studied genes was reduced in cardiac tissue of diabetic mice after 28 days of LC treatment, when compared with that in diabetic, untreated animals (p<0.05) ( Figure 4 , Table 2 ).

**Figure 4:** – Gene expression levels of chemerin (A) and CMKLRI (B) in adipose tissue of different experimental groups on days 0, 14 and 28 of LC treatment. The qRT-PCR method was used for the analysis of the relative expression of studied genes. GAPDH was used as the housekeeping gene. The level of significance between the groups at each sampling time was set at *p< 0.05, **p< 0.01, *** p< 0.001, **** p< 0.0001.

**Table 2:** – Concentration of chemerin in cardiac tissue of experimental groups. Data are shown as mean ± SD. Different lower-case letters (a,b) demonstrate significant differences between groups in each day of the experiment (p < 0.05). Different upper-case letters (A,B) demonstrate significant differences between times of treatment in each group (p < 0.05)

Cardiac tissue chemerin level (ng/mg protein)			

Days of LC treatment			

	0	14	28
Control	2.47 ± 0.42^aA^	2.56 ± 0.27^aA^	2.27 ± 0.22^aA^
Diabetic	3.01 ± 0.31^aA^	4.97 ± 0.48^bB^	5.51 ± 0.41^bB^
Diabetic + LC	2.81 ± 0.16^aA^	4.42 ± 0.38^bB^	3.13 ± 0.29^cA^
Control + LC	2.32 ± 0.38^aA^	2.67 ± 0.33^aA^	2.37 ± 0.28^aA^

*LC: l-carnitine.*

Regarding adipose chemerin and CMKLR1 mRNA and protein levels, a three-way ANOVA analysis showed a main effect of sampling time (chemerin; p = 0.0008, CMKLR1 p = 0.0001), LC treatment (chemerin; p = 0.0003, CMKLR1 p = 0.0001), diabetic condition (chemerin; p = 0.0003, CMKLR1 p = 0.0001) and interaction between three factors (chemerin; p = 0.0007, CMKLR1 p = 0.0001). It was observed that animals that received HF/HC supplemented diet had higher protein and mRNA levels of chemerin and CMKLRI in adipose tissue on days 7 , 14 and 28 after the final feeding when compared with animals that received a normal diet (p<0.05) ( Figure 5 A-B , Table 3 ). The treatment of diabetic mice with LC for 14 or 28 days resulted in a significant reduction in chemerin protein and mRNA levels in adipose tissue of diabetic animals when compared with untreated, diabetic mice (p<0.05) ( Figure 5A , Table 2 ). Animals that received LC for 14 days showed no significant change in CMKLRI expression (p>0.05), while those that were treated with LC for 28 days had a lower expression of CMKLRI when compared with untreated, diabetic mice (p<0.05) ( Figure 5B ).

**Figure 5:** – Gene expression levels of chemerin (A) and CMKLRI (B) in cardiac tissue of different experimental groups on days 0, 14 and 28 of LC treatment. The qRT-PCR method was used for the analysis of the relative expression of studied genes. GAPDH was used as the housekeeping gene. Data are shown as means ± SD. The level of significance between groups at each sampling time was set at *p< 0.05, **p< 0.01, *** p< 0.001, **** p< 0.0001.

**Table 3:** – Concentration of chemerin in adipose tissue of different experimental groups. Data are presented as mean ± SD. Different lower-case letters (a,b) demonstrate significant differences between groups in each day of the experiment (p < 0.05). Different upper-case letters (A,B) demonstrate significant differences between times of treatment in each group (p < 0.05)

Adipose tissue chemerin level (ng/mg protein)			

Days of LC treatment			

	0	14	28
Control	7.62 ± 2.58^aA^	8.11 ± 1.32^aA^	6.49 ± 1.07^aA^
Diabetic	13.22 ± 3.61^bA^	14.78 ± 2.59^bA^	16.72 ± 97^bB^
Diabetic + LC	12.64 ± 2.09^bA^	10.36 ± 1.78^cB^	10.97 ± 3.12^bB^
Control + LC	6.82 ± 1.94^aA^	7.09 ± 2.06^aA^	7.44 ± 1.19^aA^

*LC: l-carnitine.*

Regarding serum IL1-β and TNF-α levels, a three-way ANOVA analysis showed a main effect of sampling time (IL1-β; p = 0.0004 and TNF-α; p = 0.0002), treatment *(* IL1-β; p p = 0.0002 and TNF-α; p = 0.0002) diabetic condition (IL1-β; p = 0.0005, TNF-α p = 0.0001) and interaction between the three factors (IL1-β; p = 0.0008, TNF-α p = 0.0002). Serum IL1-β and TNF-α levels were significantly increased in the diabetic group when compared to the control group on days 14 and 28 after induction of diabetes (p <0.05) ( Tables 4 and 5 ). The treatment of diabetic mice with LC for 14 days had no significant effects on serum levels of IL-1β and TNF-α compared to untreated diabetic mice (p>0.05). However, four weeks after treatment with LC, serum levels of both inflammatory factors were significantly reduced, when compared to untreated diabetic mice.

**Table 4:** – Serum concentration of IL1-β in different experimental groups. Data are presented as mean ± SD. Different lower-case letters (a,b) demonstrate significant differences between groups on each day of the experiment (p < 0.05). Different upper-case letters (A,B) demonstrate significant differences between times of treatment in each group (p < 0.05)

Serum TNF-α (pmol/L)			

Days of LC treatment			

	0	14	28
Control	0.71 ± 0.19^aA^	0.65 ± 0.10^aA^	0.75 ± 0.09^aA^
Diabetic	0.65 ± 0.11^aA^	3.92 ± 0.38^bB^	7.10 ± 0.68^bC^
Diabetic + LC	1.33 ± 0.16^bA^	3.34 ± 0.50^bB^	3.94 ± 0.43^cB^
Control + LC	0.78 ± 0.18^aA^	0.98 ± 0.16^aA^	0.57 ± 0.11^dB^

*LC: l-carnitine.*

**Table 5:** – Serum concentration of TNF-α in different experimental groups on days 0, 14 and 28 of LC treatment. Data are presented as mean ± SD. Different lower-case letters (a,b) demonstrate significant differences between groups in each day of the experiment (p < 0.05). Different upper-case letters (A,B) demonstrate significant differences between times of treatment in each group (p < 0.05)

Serum IL1-β (pmol/L)			

Days of LC treatment			

	0	14	28
Control	0.93 ± 0.11^aA^	1.02 ± 0.14^aA^	0.89 ± 0.31^aA^
Diabetic	1.23 ± 0.33^aA^	2.96 ± 0.12^bB^	5.78 ± 0.21^bB^
Diabetic + LC	1.33 ± 0.26^aA^	2.23 ± 0.27^bB^	3.46 ± 0.12^cC^
Control + LC	1.07 ± 0.24^aA^	0.98 ± 0.19^aA^	0.82 ± 0.41^aA^

*LC: l-carnitine.*

## Discussion

In the current study, the effect of LC supplementation on the expression of chemerin and its receptor in adipose and cardiac tissues of experimentally-induced obese and insulin resistant mice were studied. In accordance with previous studies using rodent models of type 2 diabetes, our results showed that a five week-consumption of a HF/HC supplemented diet, together with injection of a single low dose of STZ in mice resulted in insulin resistance and obesity, which was characterized by hyperglycemia, hyperinsulinemia and increased HOMA-IR and body weight.^16 , 18 , 19^ It was observed that the HF/HC diet supplementation for five weeks resulted in hyperinsulinemia, with no obvious change in blood sugar levels, while after two weeks being fed a high calorie diet, the animals showed serum symptoms of insulin resistance, including hyperinsulinemia and hyperglycemia. These findings suggest that the serum metabolic alterations induced by this treatment were more similar to those of type 2 diabetes than of type 1 diabetes.

Our results showed that expression of chemerin and its receptor, CMKLRI, in adipose and cardiac tissues of diabetic mice were increased after induction of experimental diabetes. It was also observed that diabetic animals had higher serum levels of chemerin when compared with healthy animals. This finding suggests that simultaneous changes that occur in the levels of chemerin and its receptor in the diabetic state exacerbates the function of this hormone in the target tissues and this may play an important role in the development of functional disturbances of adipose and cardiac tissues in the diabetic status. The bulk of human and animal data supports a relationship between chemerin, obesity and metabolic syndrome, a cluster of metabolic disorders that increase the risk of CVDs. In accordance with our results, human and animal studies reported the parallel findings that obese and diabetic animals have elevated circulating levels of chemerin compared with healthy or lean subjects or animals.^22 , 23^ Several possible mechanisms may be assumed about the effects of increased expression of chemerin and CMKLRI on the initiation and progression of insulin resistance in diabetic animals. It is now known that inflammation has an important role in adipose tissue insulin resistance and cardiac muscle dysfunction.^24^ Obesity is correlated with a significant increase in the production of proinflammatory cytokines, such as IL1β and TNFα, which can induce insulin resistance and cell death in the cardiac muscle.^25 , 26^ Our findings showed the presence of increased serum levels of IL1β and TNFα following diabetes induction. Serum chemerin levels correlate with levels of proinflammatory cytokines, such as TNF-α, IL1β and IL-6.^6 , 7 , 25^ Chemerin also has adverse effects on cardiomyocyte by induction of caspase-9 and Akt-mediated cell apoptosis.^10^ Taken together, it is concluded that the observed overexpression of chemerin and its receptor in adipose and cardiac tissues of diabetic mice may have an important role in the progression of insulin resistance and cardiac dysfunctions in the diabetic status.

Our results showed that LC administration for 4 weeks can attenuate the expression of chemerin and its receptor in adipose and cardiac tissues of diabetic mice. These alterations were associated with an improvement in insulin resistance symptoms and a reduction in serum chemerin and some inflammatory factors’ levels. According to the results of the current study, previous studies showed that the use of LC leads to an increase in overall insulin sensitivity and whole body insulin-mediated glucose uptake in animals or humans with type 2 diabetes.^16 , 26^ Considerable evidence supports the fact that fatty acid oversupply and obesity leads to an ectopic accumulation of several lipid metabolites in heart muscle and possibly other tissues, which are detrimental to insulin signaling and leads to insulin resistance.^27^ Cardiac muscle cells cannot synthesize LC *de novo* and must acquire it exogenously via the carnitine/organic cation transporter 2.^28^ Based on these finding we suggest that LC supplementation in the diet may restore the LC pool and reduce lipid metabolites and lipotoxicity in cardiac and adipose tissues.

Based on our knowledge, the effects of LC on systemic chemerin level or its tissue expression in cardiac tissue of diabetic animals or humans have not been investigated so far. Our results, for the first time, show that protein and mRNA levels of chemerin and its receptor were reduced in the cardiac tissue of diabetic mice, concurrently with increased whole-body insulin sensitivity. Previous findings showed that chemerin mRNA and protein levels were elevated in the epicardial adipose tissue of patients with coronary artery disease.^29^ Serum chemerin levels are also elevated in patients with coronary artery disease and correlated with the severity and extent of coronary stenosis and several cardiometabolic parameters.^30^ It has been observed that chemerin increases apoptosis and caspase-9 activity in murine cardiomyocytes and improve the cardioprotective parameters in isolated perfused rat heart.^10 , 31^ Chemerin treatment also alters the outcome of myoblast cells, from myogenesis to adipogenesis, which is characterized by increased ROS levels and TG content in treated cells.^32^ Taken together, it was concluded that the reduction in chemerin levels and its receptor expression in cardiac tissue of diabetic mice after LC treatment may attenuate the adverse effect of chemerin on cardiomyocytes in diabetic animals. Further studies are needed to confirm this opinion.

Although the results of the current study showed that LC treatment can attenuate the increase of chemerin expression in target tissues, the molecular mechanisms regulating its expression remain poorly understood. Our results showed that LC treatment can reduce the serum levels of TNFα and IL1β in obese, diabetic rats. It was observed that these proinflammatory cytokines induce chemerin mRNA expression and secretion from 3T3-L1 adipocytes.^33^ Serum chemerin levels are also associated with serum levels of TNFα in obese patients.^34^ Based on these findings, the reduction in chemerin expression in diabetic mice may be due to a decrease in the secretion of inflammatory mediators. Hyperinsulinemia improvement may also be another mechanism caused by LC in reducing chemerin expression. Our results showed that LC could attenuate the increase in insulin levels in obese, diabetic mice. Because insulin can increase chemerin secretion from adipose tissue *in vitro* and in tissue explants,^35^ we suggest that insulin reduction in LC-treated animals may have a regulatory role on chemerin expression in adipose and cardiac tissues of diabetic mice.

There are some possible limitations to this study. In our study, gene expression was measured in total cardiac tissue in experimental animals. In light of the fact that various cardiac cell types, such as cardiomyocytes and cardiac fibroblasts, may have different actions on the pathogenesis of cardiac disease, our data did not allow differentiating expression profiles between the different cell types. Furthermore, differences in gene expression profiles between cardiac tissues from the right and left ventricles and atria have been confirmed, in addition to differences between cardiac cell types. Thus, we did not differentiate the gene expression between different parts of cardiac tissue in experimental animals. A further limitation is related to the fact that we measured all factors in young adult animals and within a short period of an experimental diabetes model. In this regard, it is of interest recalling that aging and long-term diabetes is by far the dominant risk factor for the development of cardiovascular diseases and the prevalence of cardiovascular diseases increases dramatically with increasing age. We did not determine whether LC treatment in aged rats or those with long-term diabetes leads to a similar downregulation of the expression of chemerin and its receptor in cardiac tissue of diabetic rats.

## Conclusion

In summary, our data showed that the consumption of a high-calorie diet resulted in obesity, insulin resistance and upregulation of chemerin and its receptor, CMKLRI, in adipose and cardiac tissues of mice. The treatment of diabetic mice with LC can attenuate symptoms of insulin resistance and suppress the overexpression of chemerin in diabetic animals. The results of the current experiment aid in the understanding of the novel regulatory effect of LC on cardiac gene expression in obesity and diabetes conditions. Further studies are needed to provide more evidence about nutrition therapy using LC for the non-pharmacological management of patients with diabetes and CVD based on the regulation of chemerin components in cardiac muscle.
